# Exploring Prokaryotic Communities in the Guts and Mucus of Nudibranchs, and Their Similarity to Sediment and Seawater Microbiomes

**DOI:** 10.1007/s00284-023-03397-8

**Published:** 2023-07-22

**Authors:** Tamara Stuij, Daniel F. R. Cleary, Ana R. M. Polónia, Sumaitt Putchakarn, Ana C. C. Pires, Newton C. M. Gomes, Nicole J. de Voogd

**Affiliations:** 1 Department of Biology, CESAM - Centre for Environmental and Marine Studies, University of Aveiro, 3810-193 Aveiro, Portugal; 2 Institute of Marine Science, Burapha University, Chon Buri, 20131 Thailand; 3 Naturalis Biodiversity Center, Marine Biodiversity, Leiden, The Netherlands; 4 Environmental Biology Department, Institute of Environmental Sciences (CML), Leiden University, Leiden, The Netherlands

## Abstract

In the present study, we compared mucus and gut-associated prokaryotic communities from seven nudibranch species with sediment and seawater from Thai coral reefs using high-throughput 16S rRNA gene sequencing. The nudibranch species were identified as *Doriprismatica atromarginata *(family Chromodorididae), *Jorunna funebris* (family Discodorididae), *Phyllidiella nigra*, *Phyllidiella pustulosa*, *Phyllidia carlsonhoffi*, *Phyllidia elegans*, and *Phyllidia picta* (all family Phyllidiidae). The most abundant bacterial phyla in the dataset were Proteobacteria, Tenericutes, Chloroflexi, Thaumarchaeota, and Cyanobacteria. Mucus and gut-associated communities differed from one another and from sediment and seawater communities. Host phylogeny was, furthermore, a significant predictor of differences in mucus and gut-associated prokaryotic community composition. With respect to higher taxon abundance, the order Rhizobiales (Proteobacteria) was more abundant in *Phyllidia* species (mucus and gut), whereas the order Mycoplasmatales (Tenericutes) was more abundant in *D. atromarginata* and *J. funebris*. Mucus samples were, furthermore, associated with greater abundances of certain phyla including Chloroflexi, Poribacteria, and Gemmatimonadetes, taxa considered to be indicators for high microbial abundance (HMA) sponge species. Overall, our results indicated that nudibranch microbiomes consisted of a number of abundant prokaryotic members with high sequence similarities to organisms previously detected in sponges.

**Supplementary Information:**

The online version contains supplementary material available at 10.1007/s00284-023-03397-8.

## Introduction

Nudibranchs are marine invertebrates of the order Nudibranchia (Phylum: Mollusca; Class: Gastropoda). They are a diverse group of organisms with approximately 2400 documented species across the globe [1]. Nudibranchs have been recorded across a wide range of habitats, from polar to tropical waters, and from depths below 1500 m to shallow coastal areas [2–4]. They are important consumers of benthic sessile organisms and prey on a variety of taxa including corals, sponges, and algae. At the nudibranch species level, however, feeding behavior can be highly specific, e.g., certain nudibranch species are known to only feed on a specific sponge species [5]. Until present, nudibranchs have mainly been of scientific interest due to their impressive array of chemical defenses, composed of toxins, and other bioactive metabolites [6].

Interestingly, as the widely studied prokaryotic symbionts of sponges fulfill important functions in secondary metabolite production [7–9], studies started to explore the microbial communities of nudibranchs and their possible relationship to bioactive metabolite production [10, 11]. Diverse bacterial communities inhabited the skin and guts of five dorid nudibranchs collected from the Red Sea, with OTUs assigned to the phyla Proteobacteria, Firmiticus, Tenericutes, and Bacteriodetes [12]. In addition to their 16S sequencing analysis, they revealed the presence of natural product biosynthetic gene clusters, specifically polyketide synthase (PKS) and non-ribosomal peptide synthetase (NRPS), in bacterial isolates obtained from their studied species, indicating that the genomes of these isolates contained gene clusters for natural product biosynthesis [12]. The detection of a diverse bacterial community with biosynthetic gene clusters could indicate that nudibranchs maintain mutualistic relationships with a variety of bacteria. In the present study, we aimed to explore the gut and mucus prokaryotic communities from different nudibranch species and compare these to prokaryotic communities from sediment and seawater. Whole specimens of seven nudibranch species along with seawater and sediment samples were collected from coral reefs in three regions of Thailand [Phuket (Andaman Sea), Pattaya, and Koh Tao (Gulf of Thailand)]. The species collected belonged to the most frequently documented families in a 10-year survey of Thai waters [2]. The specimens all belonged to the infraorder Doridoidei but included only two true dorids: *Doriprismatica atromarginata* and *Jorunna funebris.* Dorid species are characterized by an intact digestive gland and a feather-like plume of gills on their dorsal side, circling the anus [13, 14]. The other five species belonged to the family Phyllidiidae. Phyllidiids are characterized by hard notal tubercles, which cover their dorsum in often bright and contrasting colors. This family diverges from “true dorid” species with several modifications in their digestive tract and the replacement of the gill circlet by a series of ventrolateral gill leaflets [15]. All examined species were previously reported to feed on sponges [5, 14].

The aims of the present study were to (1) compare the diversity and composition of gut and mucus-associated prokaryotic communities with those found in sediment and seawater, (2) test for an association between gut and mucus-associated prokaryotic dissimilarity and host phylogeny, and identify potential nudibranch prokaryotic symbionts and closely related organisms. Given the fact that sponges are the main food source of the nudibranchs sampled in our study [2, 5, 16], insights into their prokaryotic communities can provide knowledge on the potential impact of diet on prokaryotic composition. Moreover, by comparing both nudibranch gut and mucus with environmental prokaryotic communities, we can delineate between generalist and biotope-specific prokaryotes, and assess to what extent nudibranch species identity and body compartment structure prokaryotic composition. This can provide important insights into the mechanisms of symbiont acquisition in these organisms.

## Methods

### Sample Collection and Species Identification

Samples were collected using SCUBA diving (5 to 20 m depth), from the 8th to the 21st of August 2014, from five different reef sites at three locations in Thailand (Supplementary Fig. S1, Table 1). Whole specimens were collected and subsequently preserved in 96% EtOH. All observed specimens were collected. Unfortunately, the sample sizes of most collected species were low limiting inter-species comparisons. In addition to this, sediment and seawater samples were collected from each location following previously described methods [17]. In short, the upper 5 cm of sediment was collected with a plastic disposable syringe from which the end had been cut off to facilitate sampling. Seawater was collected between the depths of 1–2 m with a 1.5 L bottle. To obtain the seawater prokaryotic community, water was filtered through a Millipore^®^ White Isopore Membrane Filter (0.22 µm pore size). The filters, sediment, and nudibranch samples were kept cool (<4 °C) immediately after collection and during transport. In the laboratory, samples were stored at − 80 °C until DNA extraction. In total, 21 nudibranch specimens were examined and identified based on morphological features. The cytochrome oxidase 1 (COI) gene was sequenced in order to compare nudibranch prokaryotic composition with host phylogeny. ~ 0.1 g of the nudibranch mantle tissue was placed at -80 ºC overnight, after which the samples were grinded with a mortar and pestle as finely as possible. Nudibranch DNA was extracted following the CTAB DNA extraction method [18, 19]. DNA was stored at – 20 °C until use. A 658 bp fragment of the cytochrome oxidase 1 (COI) gene was targeted for PCR amplification using the LCO1490 (5′-ggtcaacaaatcataaagatattgg-3′) and HCO2198 (5′-taaacttcagggtgaccaaaaaatca-3′) universal primers [20]. COI segments were amplified as described in Johnson and Gosliner [21], where a denaturation step at 94 °C for 3 min was followed by 39 thermal cycles of 30 s at 94 °C, 46 °C for 30 s, and 72 °C for 1 min. A final elongation step was carried out at 72 °C for 5 min. Purified PCR products were Sanger sequenced using the primer LCO1490 (GATC Biotech, Konstanz, Germany). Using the CO1 sequence data, a maximum likelihood phylogenetic tree was created using the R-package *phangorn* (Supplementary Fig. S2).

**Table 1:** (a) Total of nudibranch, sediment, and water samples collected with their specific locations as indicated in Supplementary Fig. S1 and (b) different nudibranch species collected, displayed by sample abbreviation; species name; family; total number of mucus samples; total number of gut samples and sample locations as indicated in Supplementary Fig. S1

(a)		
Sample type	*N*	Location
Nudibranch	21	1; 2; 3; 4; 5
Sediment	5	1; 4; 5
Seawater	7	1; 4; 5

(b)					
Abbr	Species	Family	No. of mucus	No. of gut	Location
*Da*	*Doriprismatica atromarginata*	Chromodorididae	3	2	1; 2
*Jf*	*Jorunna funebris*	Discodorididae	1	3	3; 5
*Pe*	*Phyllidia elegans*	Phyllidiidae	2	1	3
*Pt*	*Phyllidia picta*	Phyllidiidae	6	5	3
*Pc*	*Phyllidia carlsonhoffi*	Phyllidiidae	1	2	3
*Pn*	*Phyllidiella nigra*	Phyllidiidae	1	1	3; 4
*Pp*	*Phyllidiella pustulosa*	Phyllidiidae	2	2	3; 4
		Total	16	16	

### DNA Extraction and 16S rRNA Sequencing

Nudibranch mucus-associated communities were obtained by centrifuging the tubes containing the nudibranch specimens at 2700 g for 3 min at 4 °C. Next, the nudibranchs were removed after which the tubes were centrifuged at 4400 rpm for 1 h at 4 °C. The pellet obtained was resuspended with 122 μl of MT buffer, 978 μl of Sodium Phosphate buffer, and transferred to Lysing Matrix E tubes containing a mixture of ceramic and silica particles (buffer and tubes from FastDNA® SPIN Kit (MP Biomedicals). This method entails that the mucus-associated communities included exterior and mucus-associated prokaryotes. To obtain the gut communities, nudibranchs were dissected, after which the guts were removed from the specimens and placed into Lysing Matrix E tubes. 122 μl of MT buffer was added after which the gut was macerated with a glass rod; the rod was subsequently rinsed with 978 μl Sodium Phosphate buffer, which was then added to the Lysing Matrix E tube containing the sample. For the environmental samples, the whole membrane filter of the seawater samples or ± 500 mg of sediment was transferred to Lysing Matrix E tubes [FastDNA^®^ SPIN Kit (MP Biomedicals)]. The microbial cell lysis of the obtained mucus, gut, water, and sediment samples was performed in the FastPrep^®^ Instrument (Q Biogene) for 80 s at the speed of 6.0 m s^−1^. Subsequently, PCR-ready genomic DNA was isolated from all samples using the FastDNA^®^ SPIN Kit (MP Biomedicals) following the manufacturer’s instructions. Extracted DNA was eluted into DNase/Pyrogen-Free Water to a final volume of 50 μl and stored at − 20 °C until use.

Following the above, the 16S rRNA gene V3V4 variable region PCR primers 341F 5′-CCTACGGGNGGCWGCAG-3′ and 785R 5′-GACTACHVGGGTATCTAATCC-3′ [22] with a barcode on the forward primer were used in a 30 cycle PCR assay using the HotStarTaq Plus Master Mix Kit (Qiagen, USA) under the following conditions: 94 °C for 3 min, followed by 28 cycles of 94 °C for 30 s, 53 °C for 40 s, and 72 °C for 1 min, after which a final elongation step at 72 °C for 5 min was performed. Next-generation, paired-end sequencing was performed at MrDNA (Molecular Research LP; http://www.mrdnalab.com/; last checked 15 May 2021) on an Illumina MiSeq device (Illumina Inc., San Diego, CA, USA) following the manufacturer’s guidelines. Sequences from each end were joined following Q25 quality trimming of the ends, pairing, and removal of short reads (<150 bp).

Sequence analysis was carried out using QIIME, where fasta and qual files were used as input for the split_libraries.py script. Default arguments were used except for the minimum sequence length, which was set at 250 bps after removal of forward primers and barcodes. In addition to user-defined cut-offs, the split_libraries.py script performs several quality filtering steps (http://qiime.org/scripts/split_ libraries.html). For a detailed description of the sequence analysis, see [23]. In short, OTUs were selected using UPARSE with usearch7 [24]. The UPARSE sequence analysis tool provides clustering, chimera checking, and quality filtering on de-multiplexed sequences, where OTUs were clustered at 97% sequence similarity. Subsequently, representative sequences of the clustered OTUs were selected using the pick_rep_ set.py script using the ‘most_abundant’ method. Taxonomy was assigned to the representative sequences using the SILVA_132_QIIME_release database [25]. The DNA sequences generated in this study can be downloaded from the National Center for Biotechnology Information (NCBI) Sequence Read Archive (SRA): PRJNA397173, PRJNA397178, PRJNA397177. Sample metadata are listed in Supplementary Table S1.

### Statistical Analyses

The created OTU abundance table was imported into R [26] using the read.table() function. Before further analysis, OTUs unassigned at the domain level or classified as chloroplasts or mitochondria were removed and each sample was subsequently rarefied to 10,000 sequences per sample. The rarefied abundance table was used to calculate OTU richness (total OTU’s) and evenness (Pielou’s *J*), examining the most abundant higher taxa, and analyze bacterial community composition.

To study compositional variation among samples, the OTU abundance matrix was log_e_(*x* + 1) transformed and a distance matrix constructed using the Bray–Curtis index with the vegdist() function in the *vegan* package in R [27]. Variation in OTU composition among biotopes was visualized with Principal Coordinates Analysis (PCO) using the cmdscale() function in R with the Bray–Curtis distance matrix as input. Weighted averages scores were computed for OTUs on the first two PCO axes using the wascores() function in the *vegan* package.

Variation among biotopes (nudibranch mucus, nudibranch gut, seawater, sediment) was tested for significance using the adonis2() function (R-package *vegan*). In the adonis analysis, the Bray–Curtis distance matrix of OTU composition was the response variable with biotope as independent variable. We also tested for a difference in composition between gut and mucus samples, excluding environmental samples from the dataset. The number of permutations was set at 999; all other arguments used the default values set in the function. We used the partial.mantel function in *vegan* to test for significant associations between a distance matrix of nudibranch phylogeny and Bray Curtis dissimilarity distance matrices of gut and mucus samples separately. To control for the influence of spatial distance among the collected samples, the bray Curtis dissimilarity distance matrices were conditioned on a matrix of pairwise geographic distances between sample sites. The distance matrix of nudibranch phylogeny was obtained from the Cytochrome oxidase I (COI) sequences of the studied nudibranch specimens using the dist.hamming-function in the R-package *phangorn* (Supplementary Fig. S2). Finally, closely related organisms to the most abundant OTUs were identified using the NCBI BLAST command line “blastn” tool with the -db argument set to nt.

## Results

### Species Identification

**Fig. 1:**
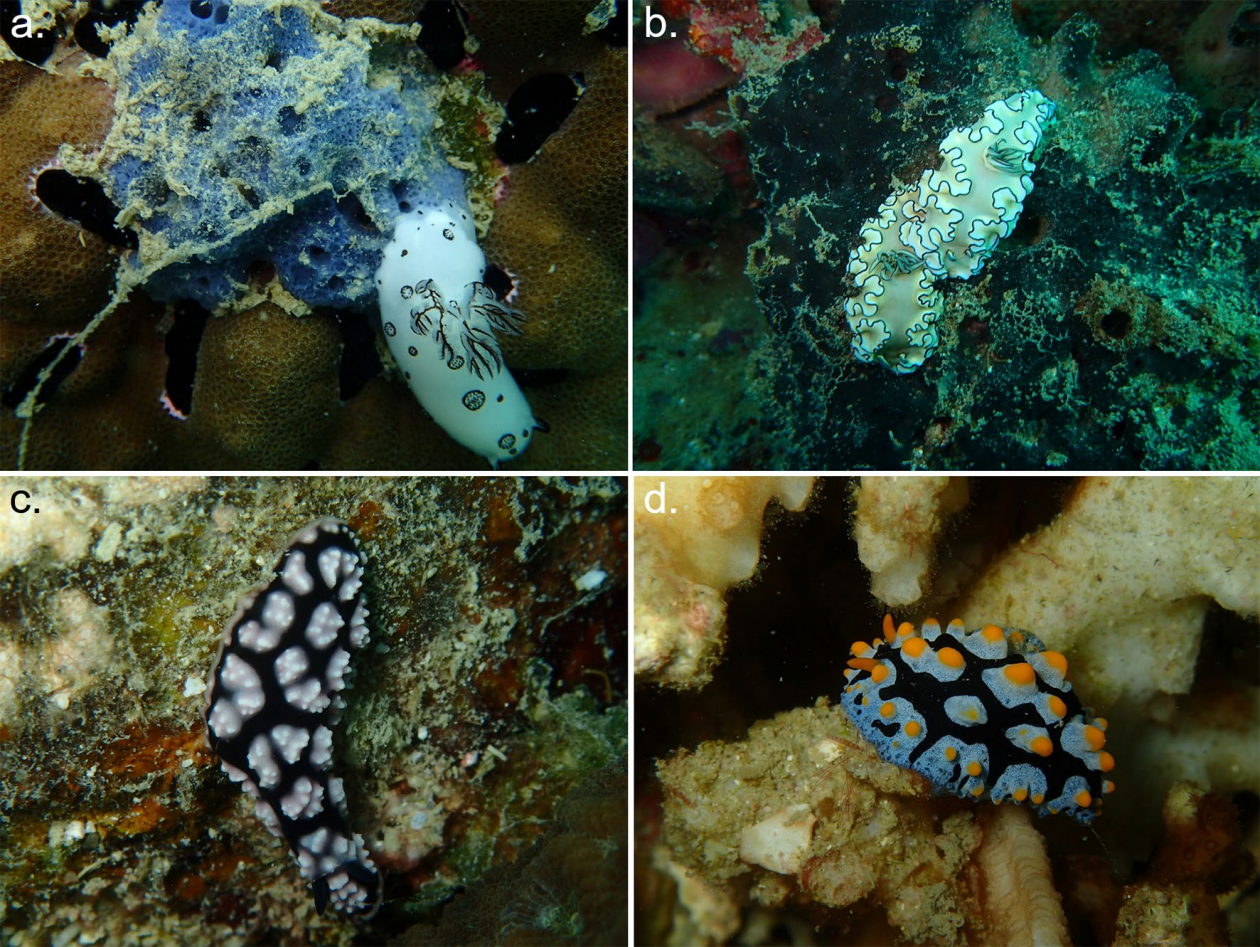
In situ photographs of selected individuals of the four genera examined in this study. **a** *Jorunna funebris* on its prey sponge *Xestospongia* sp. **b** *Doriprismatica atromarginata*. **c** *Phyllidiella pustulosa*. **c** *Phyllidia picta*

The collected specimens were identified as *Doriprismatica atromarginata* (Cuvier, 1804) (family Chromodorididae*), Jorunna funebris* (Kelaart, 1858) (family Discodorididae), *Phyllidiella nigra* (van Hasselt, 1824), *Phyllidiella pustulosa* (Cuvier, 1804), *Phyllidia carlsonhoffi* Brunckhorst, 1993, *Phyllidia elegans* Bergh, 1869 and *Phyllidia picta* Pruvot-Fol, 1957 (all assigned to the family Phyllidiidae) (Fig. 1, Supplementary Table S1 and Fig. S2). Due to the low number of sequence reads obtained from one of the *J. Funebris* samples (Jf060), this sample was excluded from further analysis. A summary of the specimens is presented in Table 1. We were not able to successfully extract high-quality DNA from both the guts and mucus of all samples, which explains the varying number of samples within a nudibranch species. Supplementary Table S1 provides additional sample details.

### Alpha Diversity and Higher Taxonomic Composition

The dataset in the present study consisted of 44 samples, 440,000 sequences, and 7469 distinct OTUs. Of the different biotopes, OTU richness was highest in sediment (a total of 5853 OTUs among the samples), followed by mucus (1908 OTUs), gut (1556 OTUs), and water samples (1148 OTUs). Excluding a single particularly rich mucus sample from a specimen of *J. Funebris* (S = 1011), rarefied richness (10,000 sequences) ranged from 86 to 449 among the nudibranch and seawater samples, whereas sediment richness ranged from 2006 to 2746 OTUs per sample. Evenness was higher in the sediment and seawater samples than nudibranch gut and mucus samples. Among nudibranch species, evenness was relatively high in mucus samples from *D. Atromarginata*, *J. Funebris,* and *P. nigra* compared to the other nudibranch samples (Supplementary Fig. S3).

**Fig. 2:**
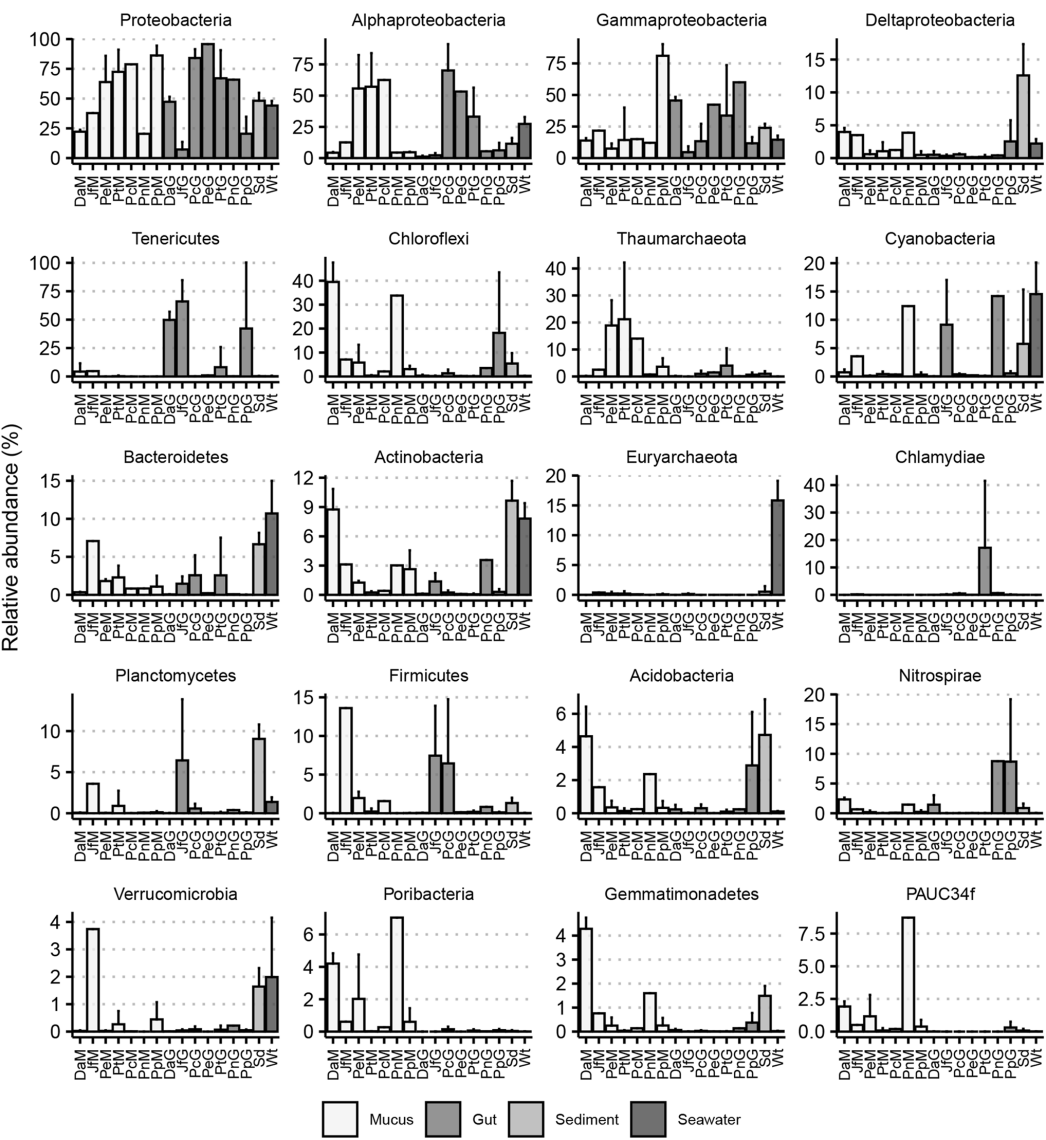
Barplots of the relative abundances of the most abundant prokaryotic phyla and three most abundant Proteobacterial classes associated with the mucus [xM and gut (xG)] of the nudibranch species: *Da* *Doriprismatica atromarginata*, *Jf* *Jorunna funebris*, *Pe* *Phyllidia elegans*, *Pt* *Phyllidia picta*, *Pc* *Phyllidia carlsonhoffi*, *Pn* *Phyllidiella nigra*, *Pp* *Phyllidiella pustulosa*, *Sd* sediment, and *Wt* seawater. Error bars represent 1 SD of the mean

In Fig. 2, the relative abundances of the 16 most abundant phyla are shown along with the three most abundant proteobacterial classes. The phylum Proteobacteria was abundant in all nudibranch and environmental samples and varied from 22.18% in *D. atromarginata* to 86.18% in *P. pustulosa* for mucus samples and from 7.24% in *J. funebris* to 95.85% in *P. elegans* for gut samples. Within the Proteobacteria, OTUs assigned to the Alphaproteobacteria class were particularly abundant in the mucus and gut samples of all *Phyllidia* species*.* Gammaproteobacteria were relatively abundant in all samples, whereas Deltaproteobacteria were most abundant in the mucus and sediment samples. The phyla Actinobacteria, Bacteriodetes, Cyanobacteria, Euryarchaeota, and Planctomycetes were more abundant in sediment and seawater samples, whereas the Tenericutes, Chloroflexi, Thaumarchaeota, Cyanobacteria, Bacteriodetes, Actinobacteria, and Firmicutes were more abundant in nudibranch samples. There was, however, considerable variation in the relative abundances of these taxa among nudibranch species. Tenericute abundance, for example, was particularly high in the guts of *J. funebris*, *D. atromarginata,* and *P. pustulosa* compared to the remaining nudibranch and environmental samples. Chloroflexi, in turn, were particularly abundant in the mucus samples of *D. atromarginata* and *P. nigra* and the gut samples of *P. picta*.

### Prokaryotic Community Variation Among Biotopes and Species

**Fig. 3:**
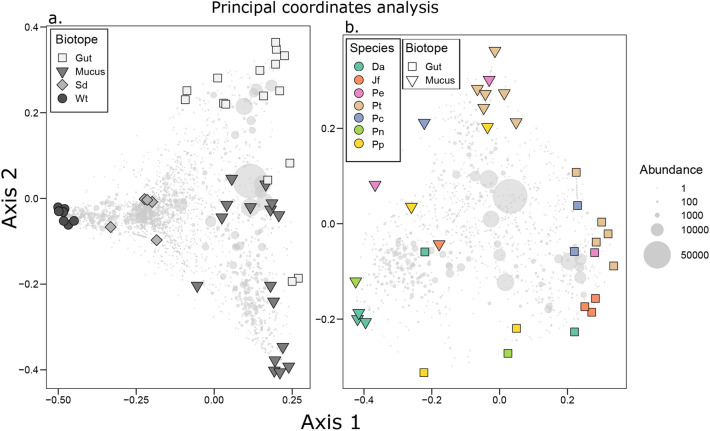
Ordination showing the first two axes of the Principal Coordinates Analysis (PCO) of prokaryote OTU composition. **a** All samples, **b** only nudibranch samples. *Da* *Doriprismatica atromarginata*, *Jf* *Jorunna funebris*, *Pe* *Phyllidia elegans*, *Pt* *Phyllidia picta*, *Pc* *Phyllidia carlsonhoffi*, *Pn* *Phyllidiella nigra*, *Pp* *Phyllidiella pustulosa*, *Sd* sediment, and *Wt* seawater. The circle size of OTUs is proportional to the abundance (number of sequences). Samples of different nudibranch species are color coded in the online version

**Fig. 4:**
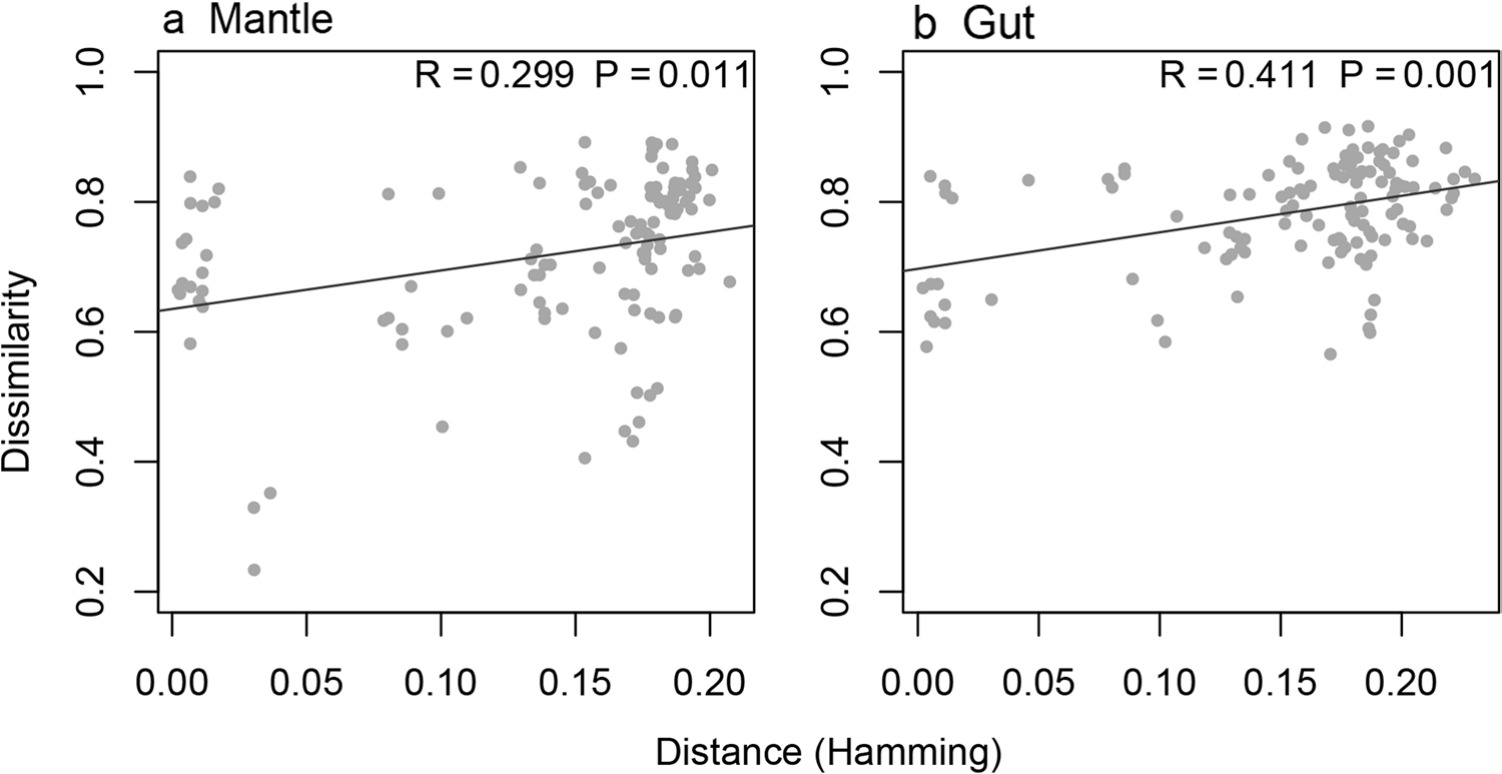
Relationship between Bray–Curtis dissimilarity (*y*-axis) and genetic distance based on COI sequence dissimilarity (*x*-axis) for (**a**) mantle and (**b**) gut samples. Mantel test results are displayed in the upper right corner of (**a**) and (**b**)

Figure 3 shows the first and second axes of the PCO analysis including nudibranch gut, mucus, sediment, and water samples. As evidenced in Fig. 3, sediment, seawater, and nudibranchs housed compositionally distinct prokaryotic communities. Figure 3a includes all samples; the factor biotope was a significant predictor of variation in composition (Fig. 3a: Adonis, *F*_3,40_ = 7.87, *P* < 0.001, *R*^2^ = 0.37). There was also a highly significant difference between mucus and gut samples, excluding sediment and seawater samples (Fig. 3b: Adonis, *F*_1,30_ = 4.97, *P* < 0.001, *R*^2^ = 0.14). After controlling for the variation due to geographic distance between collection sites, we found significant associations between Bray–Curtis and genetic dissimilarity (based on the COI data) for both gut and mucus samples (Fig. 4). Partial Mantel test: mucus: *R* = 0.236; *P* = 0.011, gut: *R* = 0.370; *P* < 0.001).

### Most Abundant OTUs and Closely related Organisms

**Fig. 5:**
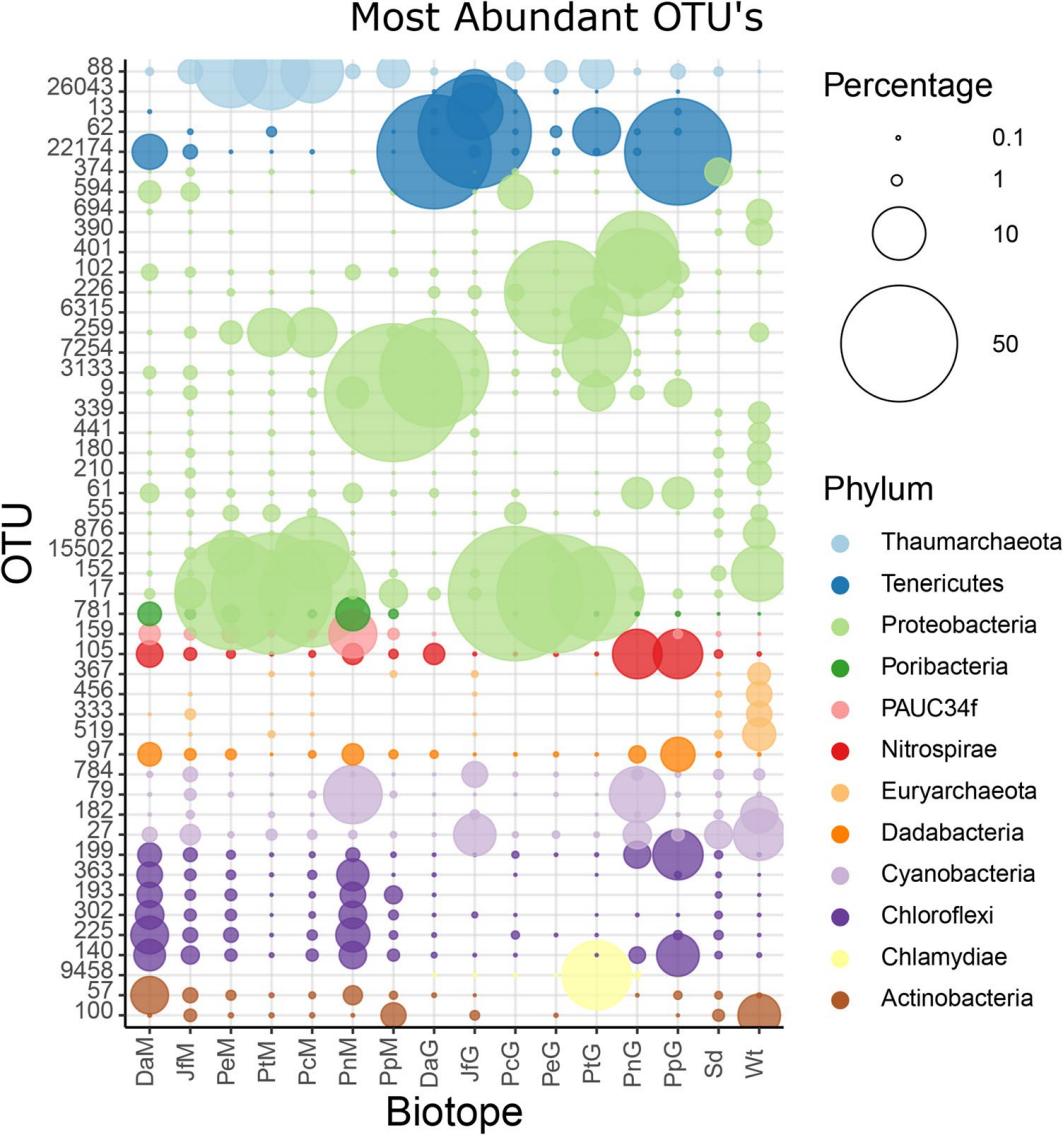
Relative abundance of the most abundant OTUs observed in mantle and gut samples. The size of the symbol is proportional to the relative abundance of the specific OTU. Gray scale or colors (online version) indicate the phyla assignment. The *y*-axis represents OTU numbers and *x*-axis nudibranch mucus (xM) and gut samples per species, sediment, and seawater. *Da* *Doriprismatica atromarginata*, *Jf* *Jorunna funebris*, *Pe* *Phyllidia elegans*, *Pt* *Phyllidia picta*, *Pc* *Phyllidia carlsonhoffi*, *Pn* *Phyllidiella nigra*, *Pp* *Phyllidiella pustulosa*, *Sd* sediment, and *Wt* seawater

The 50 most abundant OTUs are presented in Fig. 5 and Table S2. OTU-17, assigned to the family *Rhizobiaceae*, was highly abundant in the mucus and gut communities of all three *Phyllidia* species (Fig. 5) and was closely related (sequence similarity >99%) to organisms previously obtained from a coral identified as *Astrangia poculata,* a sponge identified as *Tethya californiana* and microbial mats (Table S2). OTU-88, assigned to the family Nitrosopumilaceae, was closely related to an organism previously obtained from seawater, and was also abundant in *Phyllidia* spp., particularly in the mucus-associated communities. OTU-9, assigned to the class Gammaproteobacteria, was the most abundant OTU in the mucus samples of *P. pustulosa*. OTU-1512, assigned to the family Chitinophagaceae, was abundant in the mucus-associated communities of both the *Phyllidia* and *Phyllidiella* species. Both OTUs 9 and 1512 had relatively low sequence similarity (<90%) with organisms from the NCBI database. OTU-22174, assigned to the genus *Mycoplasma*, and OTU-57, assigned to the order Acidimicrobiales, were both abundant members of the mucus prokaryotic communities of *D. atromarginata*’*s* and *J. funebris*. Furthermore, the mucus-associated communities of the dorids and the single specimen of *P. nigra* all had relatively high abundances of a number of OTUs assigned to the phyla Chloroflexi (OTUs 140, 193, 225, 302, and 363), PAUC34f (OTU-159), and Poribacteria (OTU-781) (Fig. 5). All of these OTUs were similar to organisms previously obtained from a range of sponge species including *Xestospongia testudinaria*, *Astrosclera willeyana*, *Neopetrosia chaliniformis* (as *Xestospongia exigua)*, *Rhopaloeides odorabile*, *Aplysina cauliformis*, *Amphimedon compressa*, and an endolithic community. The gut community of the dorid *D. atromarginata* was dominated by OTU-3133, assigned to the order Oceanospirillales and similar to an organism obtained from a sponge identified as *Neofibularia nolitangere*. OTU-22174 was also a dominant member of the gut community of *P. pustulosa* while OTU-62, assigned to the genus *Mycoplasma*, was the dominant member of the prokaryotic community of *J. funebris.* All OTUs assigned to the genus Mycoplasma had relatively low sequence similarity to organisms in the NCBI database (best hit had a sequence similarity of 91.3%, Table S2).

## Discussion

### General Patterns

As previously shown [17], sediment prokaryotic communities were particularly diverse in terms of richness and evenness. The biotope (sediment, seawater, and nudibranch gut and mucus samples) was a significant predictor of variation in prokaryotic community composition. This is in line with previous studies comparing the prokaryotic communities of different reef biotopes including nudibranchs [12, 16], and suggests that host identity is an important determinant of prokaryotic composition. Among the most abundant nudibranch-associated OTUs, OTUs 13, 62, 22,174, 26,043, assigned to the phylum Tenericutes, 9, 3133 and 7254, assigned to the phylum Proteobacteria, and OTU-9458, assigned to the phylum Chlamydiae, were not observed in environmental samples. OTU-88, assigned to the phylum Thaumarchaeota, and OTUs 199, 363, 193, 302, 225, 140, 9458, assigned to the phylum Chloroflexi, were enriched in nudibranch biotopes but were also present in both sediment and seawater. The occurrence of both nudibranch-specific and -enriched prokaryotes suggests that nudibranchs acquire part of their microbiome from their surroundings (horizontal transmission), and part of it via vertical transmission although this requires further verification. Vertical transmission of associated microorganisms is a feature that has also been observed in a number of other marine organisms, such as sponges [28], bivalves [29], ascidians, bryozoans, oligochaetes [30], and insects [31], and is indicative of a symbiotic relationship between the host and the vertically transferred microbe.

The variation between body compartments (mucus and gut) might be explained by the different environmental conditions apparent, e.g., in relation to acidity and temperature. Additionally, a diet rich in bioactive compounds was previously suggested to be a selective pressure within nudibranch gut microbial communities, by potentially inhibiting the growth of common gut microbes [12].

Interestingly, mucus and gut samples also tended to cluster according to host species, supporting the importance of nudibranch host identity on prokaryotic community composition. Future studies should try to obtain greater numbers of samples within species to confirm this observation. In the gut samples, prokaryotic community dissimilarity increased as a function of increasing genetic distance between pairs of nudibranch samples. A similar trend was previously observed in different mollusk species [32, 33], in addition to corals and sponges [34, 35]. Phylosymbiosis can be a result of codivergence of a host with its associated microbes [36]. Phylosymbiosis as a result of codivergence may also imply the occurrence of vertically transmitted microbes, which may eventually evolve into obligatory symbionts. Both the phylosymbiotic and species-specific patterns of the prokaryotic communities, however, may also be driven by the greater similarity in niche and diet preferences of closely related organisms compared to more distant species. Diet, in particular, is a well-documented explanation for host microbiome variation in the gut communities of various taxa [37]. Interestingly, the nudibranch species in the present study had highly specific feeding behaviors, which confounded with their phylogenetic relatedness. *D. atromarginata*, for example, preys on *Spongia* spp. [16], whereas *J. funebris* feeds exclusively on blue sponges of the genus *Xestospongia* [2]*.* Different Phyllidiidae have been found to feed on sponges of the family halichondridae and van Alphen et al. [5] noted that specimens of *P. varicosa*, *P. pustulosa*, and *P. nigra,* in reefs off West Halmahera (northern Moluccas, Indonesia), exclusively fed on the sponge *Axinyssa variabilis.* Compared to the gut communities, the relationship of the mucus-associated communities to host phylogeny was less pronounced, albeit still significant. Being more exposed to the external environment, these communities are likely to be more influenced and altered by external drivers such as seawater and sediment compared to the gut prokaryotic community. This was somewhat supported by our data, where several abundant seawater-associated OTUs (180, 210, 339, and 441) were recorded in mucus communities but were largely absent from gut communities.

### Prokaryotic Dominance and Putative Symbionts

The dominance of *Mycoplasma* (phylum Tenericutes) members observed in the dorid species and *P. pustulosa* has also been observed in the digestive systems of other invertebrates [38, 39]. Host-associated Tenericutes have undergone major genome reduction as a consequence of their intracellular lifestyle and are generally considered to be parasitic in vertebrates [39]; they acquire amino acids, nucleobases, and fatty acids from their host species. High abundances of these required substrates in the digestive tract of host organisms might favor the growth of *Mycoplasmas* [40]. Others, however, have suggested a possible beneficial role for *Mycoplasmas*, inferring they may help with digestion [41]. As an increasing amount of studies confirms their high abundance in nudibranchs [12, 17] and other mollusks [42], it will be interesting to further explore their relationships with invertebrate hosts.

The high relative abundance of OTU-17, assigned to the Rhizobiales order, in the gut and mucus-associated communities of *Phyllidia* spp. also matched findings in other invertebrates [43, 44]. In the sponge *Cymbastela concentrica*, members of the family Phyllobacteriaceae (Rhizobiales) were shown to be heterotrophic, capable of using oxygen (aerobes) or nitrate (denitrifiers) as electron acceptors and were suggested to play a role in secondary metabolite transport and/or production based on over-representation of specific COG categories [43].

Abundant OTUs in the guts of *Phyllidia* spp. were assigned to the genera *Kistimonas* and *Endozoicomonas*, both frequently associated with a variety of aquatic invertebrates such as corals, sponges, bivalves, molluscs, ascidians, echinoderms, and other nudibranch species [12, 45, 46]. Functionally, *Endozoicomonas* and *Kistimonas* spp. have been suggested to play diverse roles within their hosts including structuring the host microbiome and aiding in nutrient acquisition to disease development [46]. Also, *Endozoicomonas* were found to have a large proportion  of transposable elements incorporated into their genomes [46], and, recently, the same pattern was found for *Kistimonas*-like species in a metatranscriptomic study of the Lucinid microbiome [47]. Transposable elements have been suggested to function as agents of environmental adaptation, as they can rapidly create genetic diversity [48]. This feature matches the observations of these bacteria in a great variety of host species, fulfilling variable functional roles.

OTU-88 was an abundant archaeal member in several nudibranch species and was particularly abundant in the mucus-associated communities of *Phyllidia * spp.. This OTU was assigned to the phylum Thaumarchaeota and family Nitrosopumilaceae. Members of the *Nitrosopumilus* genus are frequently detected in sponge microbial communities and are active nitrifiers in several species [43, 49]. Moreover, a recent study compared sponge-associated MAGs (metagenome-assembled genomes) to genomes of free-living species of Thaumarchaeota, where sponge-associated *Nitrosopumilus* species revealed a functional signature of a sponge-associated lifestyle, with features related to nutrient transport and metabolism, restriction–modification, defense mechanisms, and host interactions [50].

Interestingly, our results also showed enrichment of Chlamydiae in the gut of *P. picta* (mainly in two specimens), which was largely due to the high relative abundance of OTU-9458*.* This OTU was assigned to the family Simkaniaceae. Members of this family are obligate, intracellular bacteria, found across a range of environments, and have been shown to engage in pathogenic, commensal, or mutualistic interactions with their hosts [51]. The presence in our samples indicates that this organism may have the ability to colonize the gut of *P. picta*. However, its ecological role is yet to be determined.

### The Similarity to the Sponge Microbiome

Of the 50 most abundant OTUs, blast results revealed 2 OTUs in the gut (phylum Chloroflexi) and a variety of OTUs in the mucus-associated communities (phyla Chloroflexi, PAUC34f, and Poribacteria) with high sequence similarity to organisms found in several sponge species. Chloroflexi, Poribacteria, and PAUC34f have been repeatedly recorded in sponge microbiome studies and are suggested to be indicator taxa and active members of high microbial abundance (HMA) sponge species [34]. Poribacteria isolated from the Indo-Pacific sponge *Xestospongia testudinaria*, for example, showed high expression of cell compartmentation-related genes [52]. Bacterial compartments provide confined biochemical environments, which can store volatile and toxic compounds [53], pointing towards a possible function in toxic-compound metabolism and storage. Also, whole-genome assembly studies of symbiont Chloroflexi bacteria of the sponge *Aplysina aerophoba* revealed gene clusters coding for the biosynthesis of secondary metabolites in the Chloroflexi classes Caldilineae and SAR202 [54], of which the latter was abundant in the mucus-associated communities of nudibranch species in this study. Similarity of nudibranch prokaryotic associates with their prey has been observed before. Coral feeding nudibranchs are suggested to actively transport coral bacterial associates to their cerate tips to make use of their metabolic capacities [45]. In sponge-feeding nudibranchs, it is unclear if their occurrence confers a particular functional repertoire, or if they rather represent a transient part of the nudibranch community. It should be noted that, in all our studied species, the mucus communities showed greater enrichment of sponge-associated prokaryotes compared to gut and environmental samples. Future research could focus on the temporal stability of these communities and if stable, their possible functional roles. Interestingly, in *D. atromarginata*, several Chloroflexi bacteria (OTUs 199, 363, 193, 302, 225, and 140) were enriched in both gut and mucus samples compared to environmental samples, whereas in *P. elegans*, this pattern only held true for mucus samples (only two of the six were present in the guts of *P. elegans*). All of these Chloroflexi members were closely related to organisms previously observed in association with sponges. It would also be interesting to further explore if mucus communities of sponge-feeding nudibranchs are partly composed of microbes acquired via feeding, or are only acquired via transmission from their surrounding environment, and if these transmission mechanisms differ among nudibranch species.

## Conclusion

The present study revealed significant differences in the compositions of prokaryotic communities inhabiting nudibranchs (gut and mucus), seawater, and sediment. A number of abundant nudibranch-associated OTUs were absent from sediment and seawater samples, whereas others were enriched in nudibranchs but were also present in environmental biotopes. Host-phylogeny was a significant predictor of mucus and gut-associated prokaryotic community composition, but this trend was less pronounced for mucus. We identified potential prokaryotic symbionts in gut and mucus-associated communities. For example, OTUs belonging to the genera *Mycoplasma* (OTU-13, OTU-62, OTU-22174, OTU-26043), *Kistimonas* (OTU-401, OTU-3133), and *Endozoicomonas* (OTU-226), within the families Nitrosopumilaceae (OTU-88) and Simkaniaceae (OTU-9458), and within the order Rhizobiales (OTU-17) were all related to putative symbionts found across a range of different marine host species. Moreover, the nudibranch microbiomes consisted of a number of abundant prokaryotic members with high sequence similarities to organisms previously detected in sponges. Given that all nudibranchs are mobile organisms, it is tempting to speculate that they act as a form of vector-mediated prokaryotic dispersal in marine ecosystems. Future studies should include different nudibranch species and their specific food sources (e.g., sponge species) in order to provide additional insights of the potential mechanisms of nudibranch prokaryotic acquisition and dispersion.

## Supplementary Information

Below is the link to the electronic supplementary material.

Supplementary file1 (PDF 768 KB)

Supplementary file2 (XLS 14 KB)

Supplementary file3 (XLS 15 KB)

## Data Availability

The DNA sequences generated in this study can be downloaded from the National Center for Biotechnology Information (NCBI) Sequence Read Archive (SRA): PRJNA397173, PRJNA397178, PRJNA397177. Sample metadata are listed in Supplementary Table S1.
