# Supplementary material for: Exploring Prokaryotic Communities in the Guts and Mucus of Nudibranchs, and Their Similarity to Sediment and Seawater Microbiomes
Source: Curr Microbiol. 2023 Jul 22;80(9):294. doi: 10.1007/s00284-023-03397-8 (PMC10363043; doi:10.1007/s00284-023-03397-8)
Supplement: Supplementary file 1 — Supplementary file1 (PDF 768 KB) [file 284_2023_3397_MOESM1_ESM.pdf]

Supplementary Figures

Exploring prokaryotic communities in the guts and mucus of nudibranchs, and their similarity to sediment and seawater microbiomes

Journal: Current Microbiology

Tamara M Stuij[1], Daniel FR Cleary[1], Ana RM Polónia[1], Sumaitt Putchakarn[2], Ana CC Pires[1], Newton CM Gomes[1], Nicole J de Voogd[3,4*]

[1]CESAM - Centre for Environmental and Marine Studies, Department of Biology, University of Aveiro, 3810-193 Aveiro, Portugal

[2]Institute of Marine Science, Burapha University, Chon Buri 20131, Thailand

[3]Naturalis Biodiversity Center, Marine Biodiversity, Leiden, The Netherlands

[4]Institute of Environmental Sciences (CML), Environmental Biology Department, Leiden University, The Netherlands

* To whom correspondence should be addressed:

Nicole J. De Voogd

Email: n.j.de.voogd@cml.leidenuniv.nl and/or nicole.devoogd@naturalis.nl

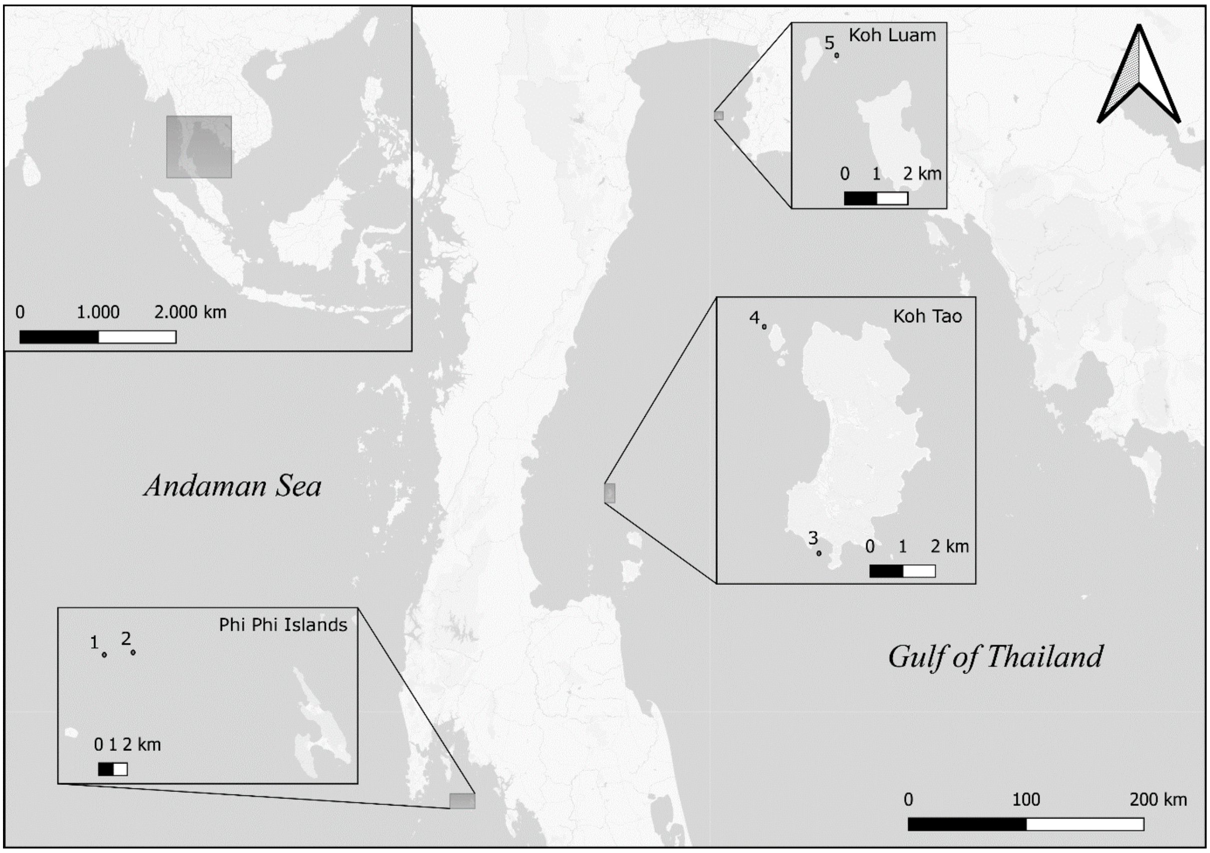

Fig. S1. Map of the study area (Andaman sea and gulf of Thailand) showing the three sample locations in higher scaled maps. Site 1 and 2: Phi Phi Islands; Site 3 and 4: Koh Tao; Site 5: Koh Luam.

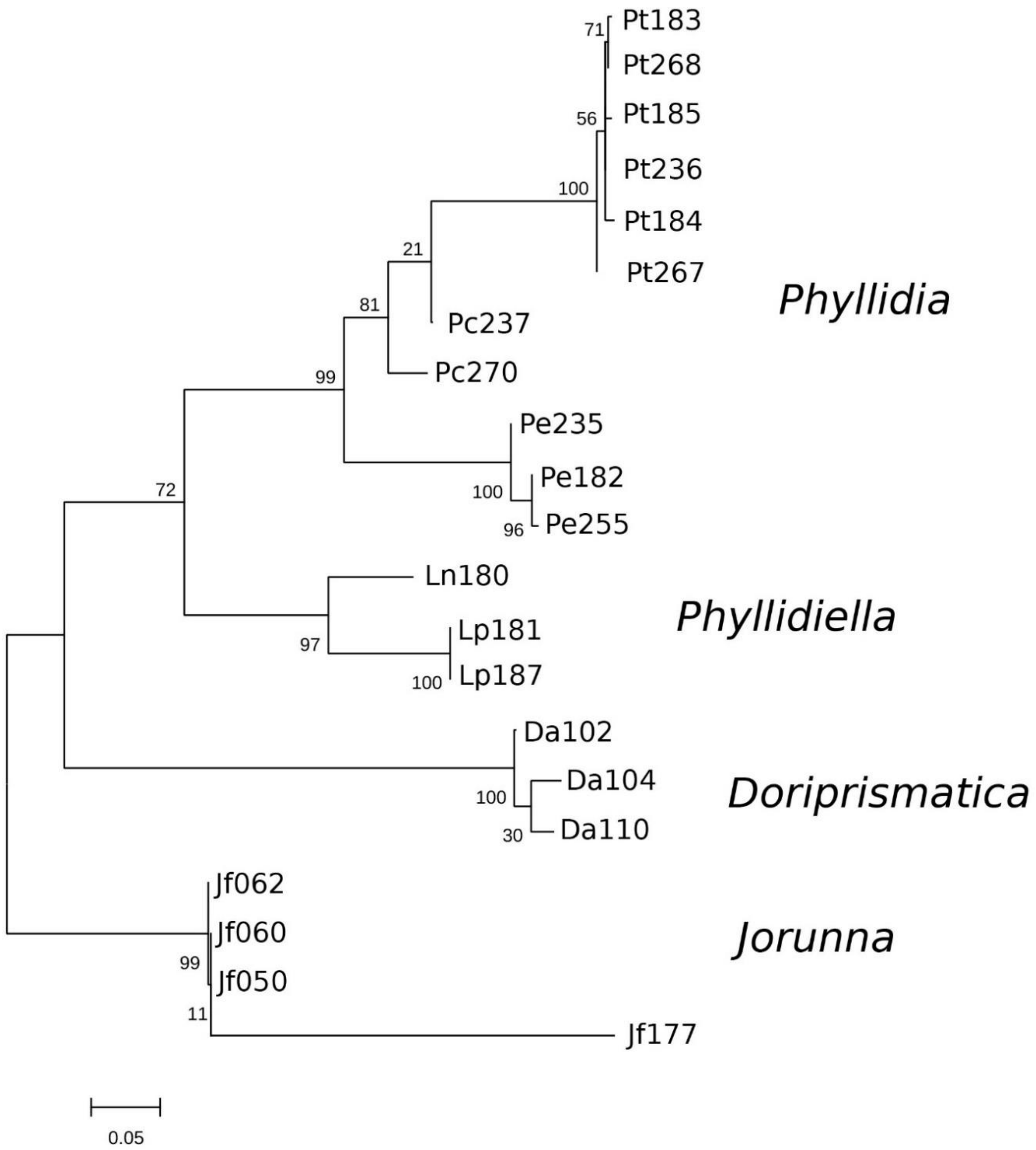

Fig. S2 Maximum likelihood phylogenetic tree based on nucleotide sequences of cytochrome oxidase I (COI) gene from the studied nudibranch specimens

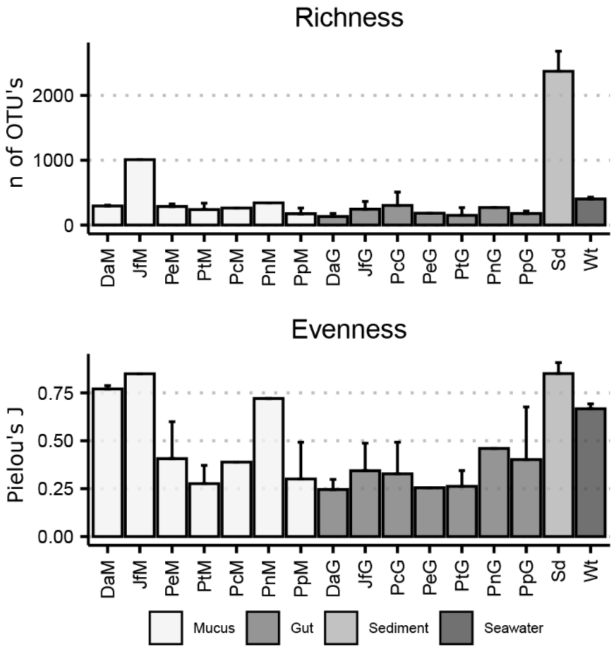

Fig. S3. Barplots of alpha diversity measures of the prokaryotic communities observed in the mucus (xM) and gut (xG) of the nudibranch species: Da, *Doriprismatica atromarginata*; Jf, *Jorunna funebris*; Pe, *Phyllidia elegans*; Pt, *Phyllidia picta*; Pc, *Phyllidia carlsonhoffi*; Pn, *Phyllidiella nigra*; Pp, *Phyllidiella pustulosa*; Sd, sediment and Wt, seawater. a Evenness. b Richness. Error bars represent one standard deviation of the mean.

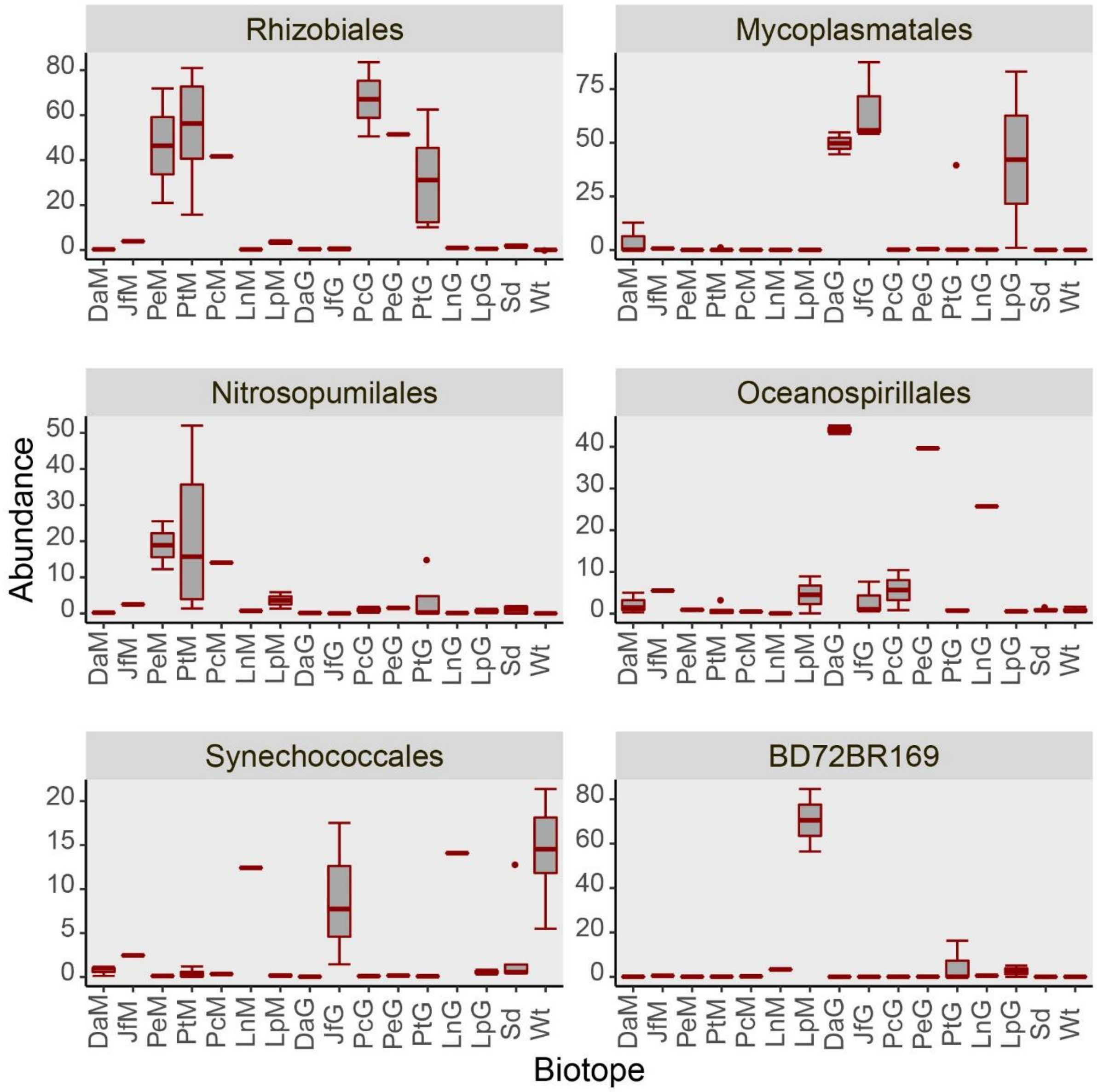

Fig. S4 Boxplots of relative abundances of the most abundant prokaryotic orders observed in the guts (xG) and mantles (xM) of the nudibranch species: Da, *Doriprismatica atromarginata*; Jf, *Jorunna funebris*; Ln, *Phyllidiella nigra*; Lp, *Phyllidiella pustulosa*; Pc, *Phyllidia carlsonhoffi*; Pe, *Phyllidia elegans*; Pt, *Phyllidia picta*; Sd, sediment and Wt, seawater
